# Toxicity reduction and immune reconstitution with adjuvant traditional Chinese medicine for postoperative ovarian cancer: a systematic review and meta-analysis

**DOI:** 10.3389/fmed.2026.1809880

**Published:** 2026-04-30

**Authors:** Yuyang Mi, Yan Wang, Qian Liu, Li Huang, Maoya Li, Min Liu

**Affiliations:** 1 Chengdu University of Traditional Chinese Medicine, Chengdu, China; 2 Gynecology Department, Hospital of Chengdu University of Traditional Chinese Medicine, Chengdu, China; 3 Pengzhou Maternal and Child Health Hospital, Pengzhou, China

**Keywords:** chemotherapy toxicity, integrative oncology, meta-analysis, ovarian cancer, traditional Chinese medicine

## Abstract

**Objective:**

To systematically evaluate the efficacy of Traditional Chinese Medicine (TCM) as an adjunctive modality in postoperative ovarian cancer management, specifically focusing on its capacity for toxicity reduction and immune reconstitution.

**Methods:**

Literature was systematically searched across six databases (China National Knowledge Infrastructure (CNKI), Wanfang Database, VIP Database, SinoMed, PubMed, and the Cochrane Library.) for literature published between January 2018 and June 2025. Randomized controlled trials (RCTs) were included if they compared integrative therapy (TCM plus conventional platinum-based chemotherapy) to conventional therapy alone in postoperative patients with ovarian cancer. The primary endpoint was the incidence of chemotherapy-related adverse events (AEs), while secondary endpoints included immune cell subsets (CD3^+^, CD4^+^, CD4^+^/CD8^+^ ratio), and Karnofsky Performance Status (KPS). Data were synthesized using random- or fixed-effect models based on heterogeneity ($I^{2}$).

**Results:**

Twenty-two RCTs (*n* = 1719) met inclusion criteria. Adjunctive TCM significantly reduced chemotherapy-related AEs (RR = 0.35, 95% CI 0.27–0.46; $I^{2}$=0%), and increased T-cell subsets: CD3^+^ levels improved (MD = 11.74, 95% CI 10.62–12.85; $I^{2}$=0%) and CD4^+^ levels increased (MD = 10.04, 95% CI 7.31–12.78; $I^{2}$=93%), whereas the CD4^+^/CD8^+^ ratio did not show a statistically significant difference (SMD = 3.22, 95% CI 2.41–4.04; $I^{2}$=90%). KPS scores were higher in the TCM group (MD = 13.06, 95% CI 11.97–14.15; $I^{2}$=93%).

**Conclusion:**

The integration of TCM with postoperative chemotherapy provided dual benefit of reducing treatment toxicity and enhancing cellular immune recovery in patients with ovarian cancer. Due to limitations in study quality and heterogeneity, further high-quality RCTs are needed to validate these findings and establish standardized TCM protocols.

**Systematci review registration:**

https://www.crd.york.ac.uk/PROSPERO/view/CRD420251242407, CRD420251242407.

## 1 Introduction

Ovarian cancer remains the most lethal challenge within gynecologic oncology. While cytoreductive surgery combined with platinum-based chemotherapy constitutes the standard of care for the majority of patients diagnosed at advanced stages (1), this aggressive therapeutic approach introduces a critical clinical paradox. Although these interventions are essential for prolonging survival, they precipitate a state of postoperative immunosuppression and functional exhaustion (2). These chemotherapy-related adverse events are not merely transient side effects; they represent substantial prognostic barriers that degrade quality of life and compromise treatment adherence, ultimately undermining the very survival benefits the therapy intends to achieve (3).

The post-surgical landscape is frequently dominated by a “toxicity cascade,” characterized by delayed gastrointestinal recovery, myelosuppression, and neurotoxicity (4). When these cumulative physiological burdens necessitate dose reductions or the premature discontinuation of chemotherapy (5), the window for curative control narrows significantly. Consequently, there is an urgent unmet need for integrative strategies that can simultaneously mitigate treatment-related toxicities and accelerate the restoration of immune competence (6), thereby ensuring patients can tolerate the full course of oncologic therapy.

Traditional Chinese Medicine (TCM), an integral component of China’s medical system, has long been used in the management of gynecologic malignancies, perioperative rehabilitation (7), and cancer-related immune modulation, including decotions/granules/patent medicines, acupuncture, etc. In TCM theory, ovarian cancer is categorized as zhengjia (abdominal mass) and jiju (accumulation), conditions characterized by qi and blood deficiency, spleen dysfunction, and the coexistence of stasis and toxin (8). Therapeutic principles emphasize strengthening vital qi, invigorating the spleen, promoting blood circulation, and eliminating toxins (9). Contemporary clinical studies suggest that TCM may enhance immune parameters, such as CD3^+^ and CD4^+^ T-cell subsets, natural killer cell activity, and immunoglobulin levels, and facilitate the recovery of postoperative gastrointestinal function. Additionally, TCM has been shown to alleviate clinical symptoms, improve the Karnofsky Performance Status score, enhance quality of life, and reduce the incidence of chemotherapy-related adverse reactions (10, 11).

However, the current evidence base remains limited, largely due to small sample sizes, single-center randomized controlled trials with heterogeneous intervention protocols, and inconsistent outcome indicators (12). Although several systematic reviews and meta-analyses—including those conducted by Li (13) and colleagues—have evaluated the efficacy and safety of Chinese herbal medicine in ovarian cancer patients receiving cytoreductive surgery and adjuvant chemotherapy, most have focused primarily on survival outcomes or adverse reactions. Immunologic outcomes and comprehensive postoperative recovery indicators have been insufficiently reported, and many reviews did not incorporate randomized controlled trials published after 2020.

To address these gaps, the present study conducted a systematic review and meta-analysis of randomized controlled trials evaluating TCM in combination with conventional therapy for postoperative ovarian cancer. Building on previous evidence, this review specifically includes patients with histologically confirmed ovarian malignancies who underwent radical or cytoreductive surgery and subsequently received platinum-based chemotherapy, and comprehensively examines the effects of combined TCM therapy on chemotherapy-related toxicities, immune function, and functional status. By extending the search period to 2025 to focus on the data from recent years and applying standardized methodological procedures, this study aims to provide more robust, contemporary evidence to inform integrated postoperative treatment strategies and support the design of future high-quality clinical trials.

## 2 Methods

### 2.1 Literature search strategy

A comprehensive search strategy combining electronic database searching and manual screening was employed to identify eligible studies. The following databases were searched from January 2018 to June 2025: China National Knowledge Infrastructure (CNKI), Wanfang Database, VIP Database, SinoMed, PubMed, and the Cochrane Library. Both Medical Subject Headings (MeSH) and free-text terms were used, with key terms including “ovarian cancer,” “ovarian neoplasms,” “traditional Chinese medicine,” “Chinese herbal medicine,” “integrative therapy,” “chemotherapy,” “platinum-based regimen,” “randomized controlled trial” and “clinical trial” along with their corresponding Chinese terms for domestic databases. Boolean operators (AND, OR) were applied to ensure adequate search sensitivity and specificity. To further enhance completeness, the reference lists of included studies and relevant reviews were manually screened to identify additional eligible trials. The study protocol was prospectively registered in PROSPERO (registration no. CRD420251242407).

### 2.2 Inclusion and exclusion criteria

#### 2.2.1 Inclusion criteria

Eligible studies were required to meet all of the following criteria:

(1) *Study design*: Prospective randomized controlled trials published in Chinese or English.
(2) *Participants*: Women with histopathologically confirmed ovarian malignancies who were in the perioperative, postoperative, or chemotherapy phase, including patients receiving cytoreductive/radical surgery followed by platinum-based chemotherapy, as well as ovarian cancer survivors undergoing symptom or quality-of-life management during or after systemic therapy. No restrictions were imposed regarding age, FIGO stage, or pathological subtype.
(3) *Interventions*: The control group received conventional management appropriate to the clinical setting (e.g., surgery and/or platinum-based chemotherapy, standard perioperative care, usual supportive care, or sham/placebo when reported). The experimental group received the same conventional management plus a clearly defined Traditional Chinese Medicine (TCM)–related intervention, including Chinese herbal decoctions/granules/patent medicines, TCM injections (e.g., Shenqi Fuzheng injection), or TCM non-pharmacologic modalities such as acupuncture, moxibustion, or auricular acupressure with specified duration and frequency.
(4) *Outcomes*: Studies reporting at least one of the following: incidence of chemotherapy-related adverse events; immune function indicators (CD3^+^, CD4^+^, CD4^+^/CD8^+^ ratio); Karnofsky Performance Status (KPS) score; or quality-of-life (QOL) score; Sufficient numerical data (means ± SD, or event counts) were required for effect-size calculation.
(5) *Baseline comparability*: Comparable baseline characteristics between treatment groups without evidence of major selection bias.
(6) Published between January 2018 and June 2025.

Systematic reviews, case reports, single-arm studies, and trials lacking a control group were not eligible.

#### 2.2.2 Exclusion criteria

Studies were excluded if they met any of the following conditions:

(1) Non-randomized studies, single-arm trials, pre–post studies, case series, review articles, case reports, conference abstracts, or dissertations.
(2) Interventions in which TCM was not clearly defined or was also administered in the control group, preventing accurate comparison.
(3) Missing or unextractable outcome data, with unsuccessful attempts to obtain missing information from authors.
(4) Duplicate publications or studies with overlapping samples (the most complete version was retained).
(5) Participants with additional malignancies or severe organ dysfunctions when ovarian cancer-specific outcomes could not be reliably assessed.
(6) Studies that did not clearly involve malignant ovarian tumors or included multiple primary cancers without distinguishable ovarian cancer–specific outcomes.
(7) Trials with incomplete reporting of key endpoints—such as chemotherapy-related adverse events, or immune parameters, preventing estimation or reconstruction of effect sizes.

Studies were excluded if they lacked extractable outcome data, did not clearly diagnose ovarian cancer, or failed to distinguish the independent effect of TCM from other concomitant interventions.

### 2.3 Data extraction

Two reviewers independently extracted data using a standardized data extraction form. Extracted information included:

(1) General study information: first author, publication year, study region, and publication type.
(2) Study characteristics: randomization method, allocation concealment, blinding procedures, and duration of follow-up.
(3) Participant characteristics: sample size (experimental/control), age, FIGO stage, pathological subtype, and baseline comparability.
(4) Intervention details: chemotherapy regimen and cycles in the control group; type, composition, dosage, and duration of TCM intervention in the experimental group.
(5) Outcome indicators: adverse event counts; immune markers (CD3^+^, CD4^+^, CD4^+^/CD8^+^ ratio); KPS score; and QOL score.

Any discrepancies between reviewers were resolved through discussion or consultation with methodological experts.

### 2.4 Assessment of methodological quality

Two reviewers independently assessed the methodological quality of included studies using the Cochrane Risk of Bias tool. The following domains were evaluated:

(1) Random sequence generation;
(2) Allocation concealment;
(3) Blinding of participants and personnel;
(4) Blinding of outcome assessors;
(5) Completeness of outcome data;
(6) Selective outcome reporting;
(7) Other potential sources of bias.

Each item was categorized as “low risk,” “high risk” or “unclear risk.” Disagreements were resolved by discussion or adjudication by a third reviewer. Detailed assessments are presented in a summary table, with overall risk-of-bias visualization shown using traffic-light plots.

### 2.5 Statistical analysis

Statistical analyses were conducted using RevMan 5.4. For dichotomous variables, risk ratios (RRs) with 95% confidence intervals (CIs) were calculated. For continuous variables, mean differences (MDs) or standardized mean differences (SMDs) with 95% CIs were computed. Heterogeneity was quantified using the χ^2^ test and the *I^2^* statistic. A fixed-effects model was applied when *p* ≥ 0.10 and *I^2^* ≤ 50%; otherwise, a random-effects model was used. When ≥10 studies were available, publication bias was evaluated visually using funnel plots.

For outcomes with substantial heterogeneity (*I^2^* > 75%), meta-regression analyses were performed using the restricted maximum likelihood (REML) method to explore potential sources of heterogeneity. Covariates for the regression model were selected based on clinical relevance and data availability from the included studies.

We performed a leave-one-out sensitivity analysis to explore the source of high heterogeneity. Studies that contributed to significant heterogeneity (*I^2^* > 50%) were excluded iteratively, and the meta-analysis was re-run to assess the robustness of the result. A fixed-effect model was used for the updated analysis after heterogeneity was eliminated (*I^2^* < 50%).

## 3 Results

### 3.1 Literature search and study selection

**Figure 1:**
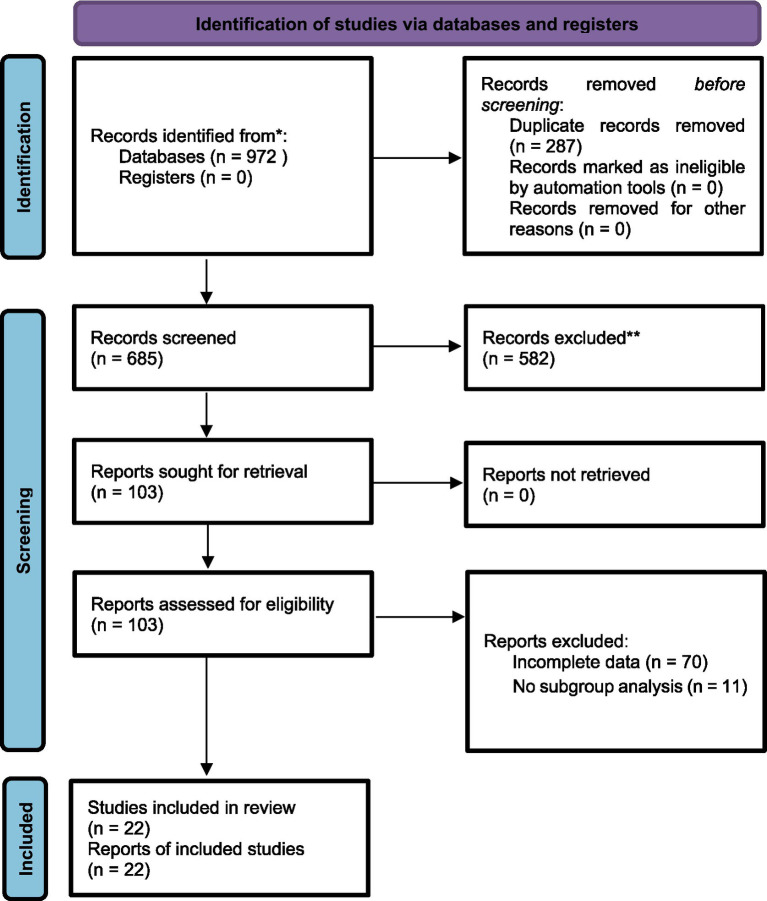
Literature screening process.

The initial systematic search across six major databases yielded a total of 972 potential citations. The source distribution included 303 records from CNKI, 387 from Wanfang, 16 from VIP, 16 from SinoMed, 226 from PubMed, and 11 from the Cochrane Library. Following the removal of 287 duplicates using Endnote X8, 685 records were subjected to a hierarchical screening process. Initial screening by title and abstract led to the exclusion of 582 records deemed irrelevant, non-clinical, or unrelated to ovarian malignancy. The remaining full-text articles were rigorously assessed against inclusion criteria; 81 were subsequently excluded due to non-randomized designs, intervention misalignment, data insufficiency, or inappropriate outcome reporting. Ultimately, 22 randomized controlled trials (RCTs) satisfied all eligibility criteria and were included in the quantitative synthesis. The detailed screening process is illustrated in the PRISMA flow diagram (Figure 1).

### 3.2 Characteristics of included studies

The final dataset comprises 22 RCTs (14–35) published between January 2018 and June 2025, representing a contemporary evidence base. While the geographic distribution is predominantly anchored in China, the inclusion of data from Japan and other regions reflects a broadening investigation into TCM-related integrative modalities. The cumulative cohort consisted of women with histologically confirmed ovarian malignancies, managed across perioperative, postoperative, and adjuvant chemotherapy settings.

Regarding trial design, the interventional arm consistently featured an integrative approach in which patients received standard-of-care chemotherapy combined with specific TCM protocols, primarily herbal decoctions, granules, or proprietary Chinese medicines, and a subset of trials incorporated acupuncture or moxibustion. Conversely, control arms were strictly limited to identical conventional chemotherapy regimens without TCM adjunctive therapy. Sample sizes across the included studies ranged from 30 to 80 participants. Crucially, baseline demographic and clinical characteristics, including age, FIGO stage, and pathological subtypes, demonstrated satisfactory comparability between groups.

The outcome assessment followed a structured hierarchy: the primary endpoint was the incidence of chemotherapy-related adverse events. Secondary endpoints evaluated the recovery of immune competence (specifically CD3^+^, CD4^+^, CD4^+^/CD8^+^ ratio), functional status (KPS scores), and quality of life. Detailed characteristics and the methodological quality assessment of all included studies are summarized in Table 1.

**Table 1:** General characteristics and quality assessment of included studies.

First author (Year)	Sample size (I/C)	TCM intervention (Formula/Mode)	Chemotherapy	Control group	Duration (week)	Primary outcome measures	Risk of bias (D1/D2/D3/D4/D5)
Li Y, 2020 (14)	115/115	Yiqi Huoxue Jiedu decoction (oral, TCM formula)	Platinum-based	Platinum-based	9	CD3^+^, CD4^+^, CD4^+^/CD8^+^ Ratio	L/L/H/S/S
Mao F, 2021 (15)	35/35	Fuyang Yishen Prescription (TCM decoction)	Platinum-based	Platinum-based alone	9	CD3^+^, CD4^+^, CD4^+^/CD8^+^ Ratio	S/L/S/H/S
Matsumura Y, 2024 (16)	27/28	Goshajinkigan (TCM compound/Japanese Kampo)	Paclitaxel-containing	Standard care	-	Chemotherapy-Related Adverse Events	L/L/L/H/L
Li PC, 2025 (17)	9/10	Jing Si herbal tea (oral TCM preparation)	Platinum-based	Platinum-based alone	-	Adverse Events	H/L/S/L/H
Guo LR, 2023 (18)	45/45	Huangqi Zhishi decoction (oral TCM decoction)	Platinum-based + Bevacizumab	Platinum-based + Bevacizumab alone	12	CD3^+^, CD4^+^, CD4^+^/CD8^+^ Ratio, Adverse Events	S/H/L/L/L
Zick SM, 2021 (19)	30/29	Self-acupressure (Auricular point acupressure protocol)	Platinum-based (survivors/post-treatment)	Usual care	12	Adverse Events, CD4^+^, QoL	L/L/H/S/S
Ben, 2023 (20)	70/29	Acupuncture (Perioperative auricular acupuncture)	Gynecologic oncology surgery + Platinum	Standard perioperative care	12	Adverse Events, QoL	S/S/L/L/H
Li XW, 2019 (21)	35/35	Jianpi Lishui Recipe (oral TCM preparation) + Intraperitoneal IL-2	Platinum-based + Intraperitoneal IL-2	Intraperitoneal IL-2 alone	14	Adverse Events	L/S/L/H/S
Li Jing, 2022 (22)	30/30	YiQiShengSui formula (oral TCM formula) + rhG-CSF	Platinum-based	rhG-CSF alone	9	CD3^+^, CD4^+^, CD4^+^/CD8^+^ Ratio, QoL	L/L/H/H/L
Li R, 2020 (23)	31/31	Acupuncture + TCM (comprehensive TCM intervention)	Platinum-based	Platinum-based alone	12	Adverse Events, QoL	L/L/L/L/L
Yang L, 2020 (24)	60/50	Shenqi Fuzheng injection (TCM injection)	TC regimen (Paclitaxel + Carboplatin)	TC alone	9	Adverse Events, CD3^+^, CD4^+^, CD4^+^/CD8^+^ Ratio	S/H/L/L/H
Huang WJ, 2022 (25)	40/40	Wenyang Yiqi Jianpi decoction (oral TCM decoction)	Platinum-based	Platinum-based alone	12	Adverse Events, CD4^+^	H/S/L/L/S
Chen XX, 2022 (26)	40/40	Moxibustion (non-pharmacologic TCM modality)	Platinum-based (postoperative)	Standard care	12	Adverse Events, QoL	L/L/S/L/L
Zhang H, 2025 (27)	29/29	Acupuncture + TCM enema (comprehensive TCM)	Platinum-based	Platinum-based alone	-	Adverse Events	H/H/H/H/S
Kuo HC, 2018 (28)	23/24	Auricular point acupressure (seed embedding method)	Platinum-based	Standard care	-	Adverse Events, QoL	H/H/H/S/L
Li Y, 2025 (29)	41/41	Modified Yiqi Yangyin formula (oral TCM formula)	Platinum-based	Platinum-based alone	12	Adverse Events, CD3^+^, CD4^+^, CD4^+^/CD8^+^ Ratio	L/L/L/L/L
Yin BL, 2025 (30)	40/40	Modified Xiuhe formula (oral TCM decoction)	Platinum-based (postoperative)	Platinum-based alone	18	Adverse Events, CD3^+^, CD4^+^, CD4^+^/CD8^+^ Ratio	S/S/S/S/S
Tsao Y, 2019 (31)	30/30	Auricular acupressure (standardized TCM protocol)	Platinum-based	Usual care	9	Adverse Events, CD4^+^, QoL	L/S/L/S/S
Hu T, 2021 (32)	32/32	Yiqi Yangyin Formula (oral TCM formula)	Platinum-based (postoperative)	Platinum-based alone	-	Adverse Events	L/S/L/L/L
Zhang LJ, 2025 (33)	47/48	Modified Qigui Xiaozheng formula (oral TCM formula)	TC regimen	TC alone	12	Adverse Events, CD4^+^	L/S/S/H/S
Shao HO, 2023 (34)	37/37	Modified Xiuhe formula (oral TCM formula)	TC regimen (middle-to-advanced stage)	TC alone	18	Adverse Events, CD4^+^	L/S/H/H/L
Wu X, 2022 (35)	38/37	Yiqi Huoxue Jiedu Formula (oral TCM formula)	Platinum-based (platinum-resistant patients)	Platinum-based alone	-	Adverse Events	S/S/L/H/L

I/C, Intervention/Control; D1, Randomization process; D2, Allocation concealment; D3, Blinding of participants and personnel; D4, Blinding of outcome assessment; D5, Incomplete outcome data. Risk of Bias levels: L, Low risk; S, Some concerns; H, High risk. NA, Not applicable for non-pharmacological interventions. TC = Paclitaxel + Carboplatin; TP = Paclitaxel + Cisplatin.

### 3.3 Methodological quality assessment

**Figure 2:**
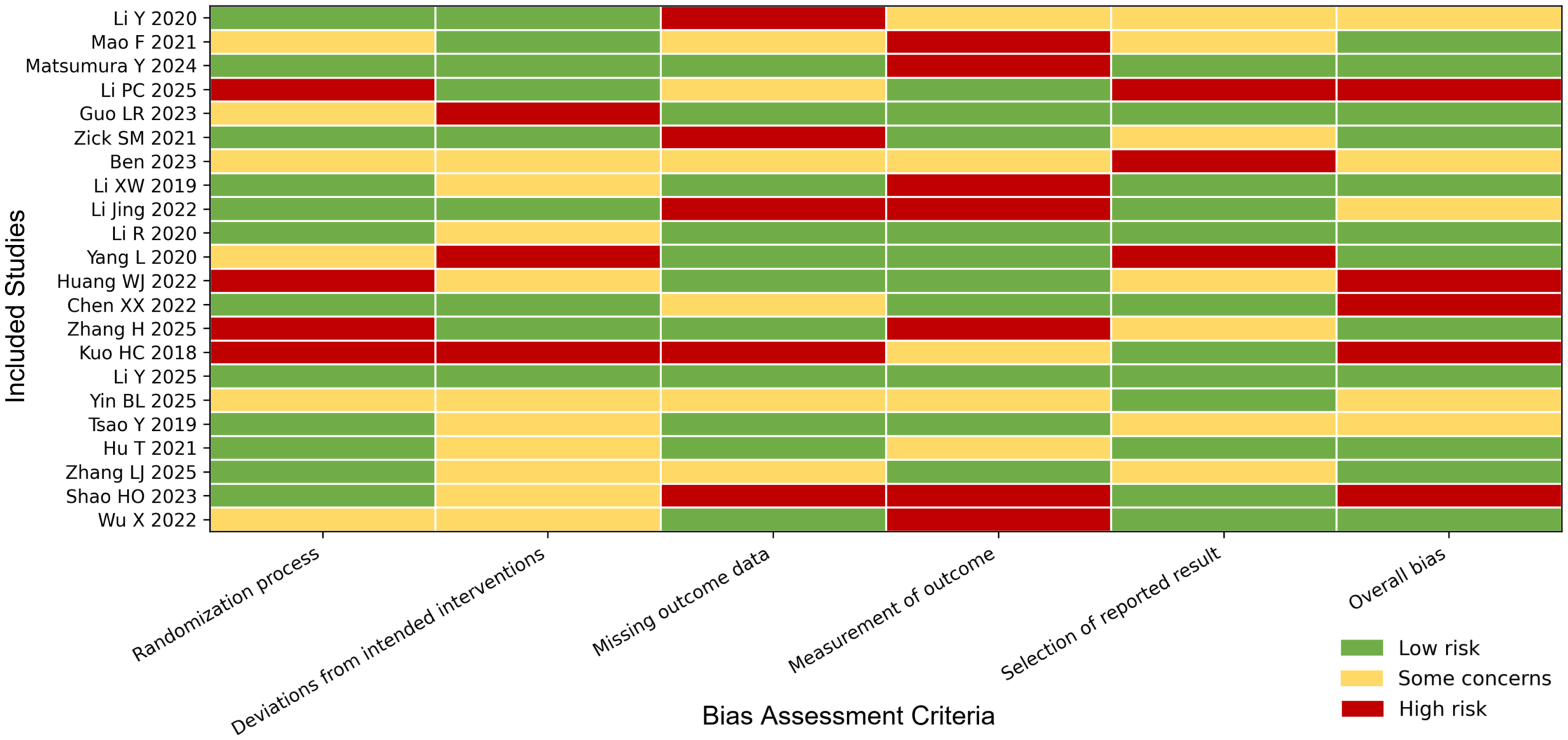
Risk of bias graph.

The methodological rigor of the included randomized controlled trials (RCTs) was critically appraised using the Cochrane Risk of Bias tool, with the aggregate risk landscape illustrated in Figure 2. While data integrity was high, as evidenced by 100% of studies being classified as low risk for incomplete outcome data, structural limitations inherent to integrative medicine trials were observed. Specifically, regarding selection bias, 10 trials demonstrated robust random sequence generation (low risk), whereas the remaining 12 presented unclear risks due to insufficient reporting detail.

Performance bias represented the most significant challenge; only five studies implemented explicit blinding protocols. The majority (17 studies) were rated as high risk in this domain, a common constraint in trials involving distinct physical interventions like herbal decoctions or acupuncture where double-blinding is logistically complex. This methodological limitation should be taken into consideration when interpreting the findings, particularly for subjective outcomes such as KPS, which may be more susceptible to bias. In contrast, objective outcomes, including adverse events and immunological indicators, are less likely to be influenced by performance bias. The limitations were balanced against the objective nature of the primary biological endpoints, mitigating the potential for subjective skew in outcome assessment.

### 3.4 Meta-analysis

#### 3.4.1 Chemotherapy-related adverse events

The mitigation of treatment-associated toxicity emerged as one of the most consistent benefits of the integrative therapeutic strategy. A total of 19 RCTs were included in the quantitative synthesis evaluating chemotherapy-related AEs.

**Figure 3:**
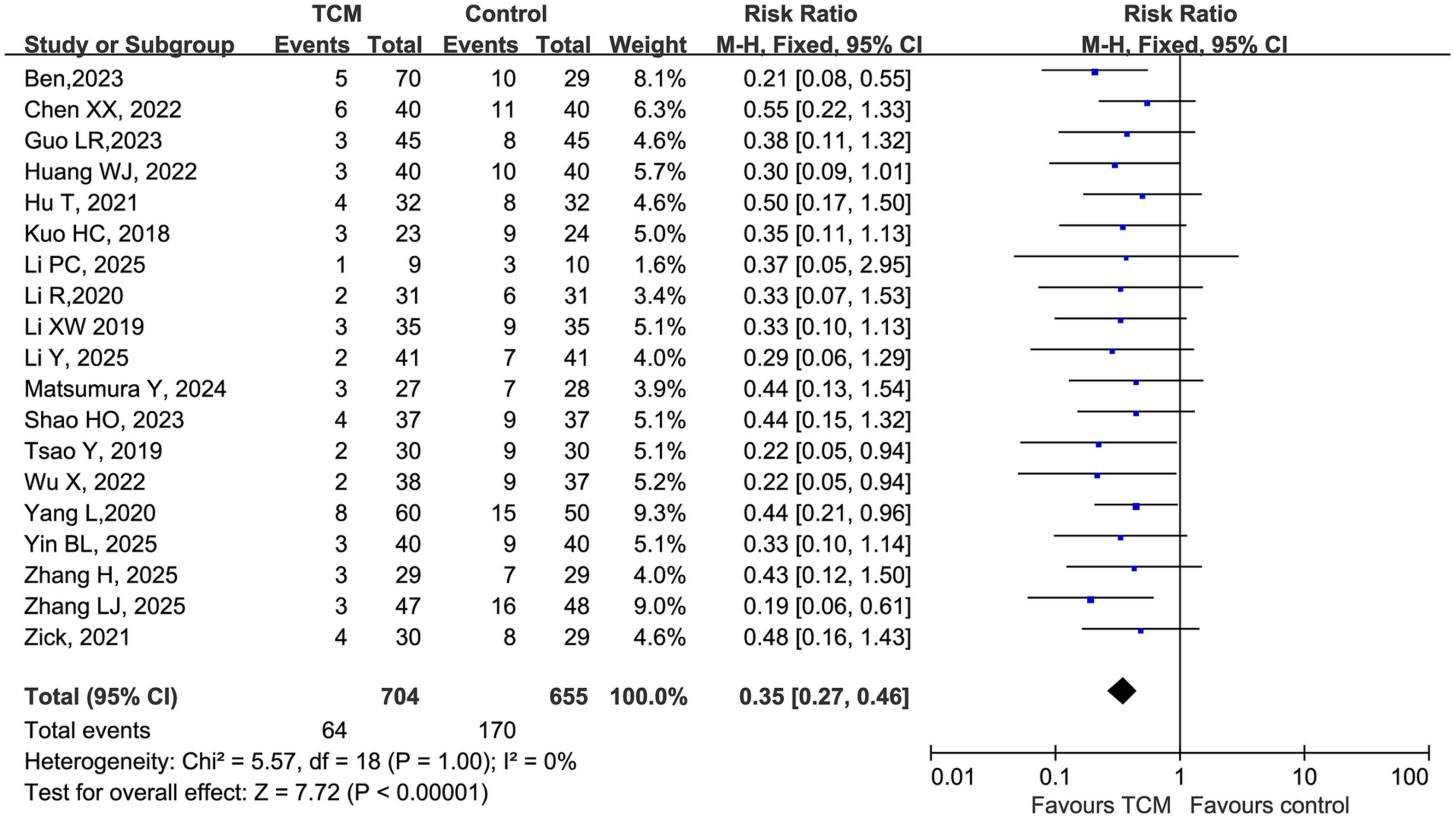
Forest plot of pooled risk ratios (RRs) comparing the incidence of chemotherapy-related adverse events between the TCM plus chemotherapy group and the chemotherapy-alone group.

Using a Mantel–Haenszel fixed-effects model, pooled analysis based on risk ratios (RRs) demonstrated that the addition of traditional Chinese medicine (TCM) to standard chemotherapy was associated with a significant reduction in the risk of chemotherapy-related AEs compared with chemotherapy alone (RR = 0.35, 95% CI 0.27–0.46; *Z* = 7.72, *p* < 0.00001; Figure 3). This corresponds to an approximate 65% relative risk reduction in overall AE incidence among patients receiving adjunctive TCM. No statistically significant heterogeneity was observed across studies ($I^{2}$ = 0%, *χ^2^* = 5.57, *p* = 1.00), indicating a high level of consistency across different trials and clinical settings.

#### 3.4.2 Immune function: CD3^+^, CD4^+^, CD4^+^/CD8^+^ ratio

To investigate the biological mechanisms associated with clinical recovery, we analyzed data on key immune effectors. The integrative therapy markedly improved cellular immunity, significantly increasing the counts of CD3^+^ and CD4^+^ T cells, which are essential for adaptive immune responses.

**Figure 4:**
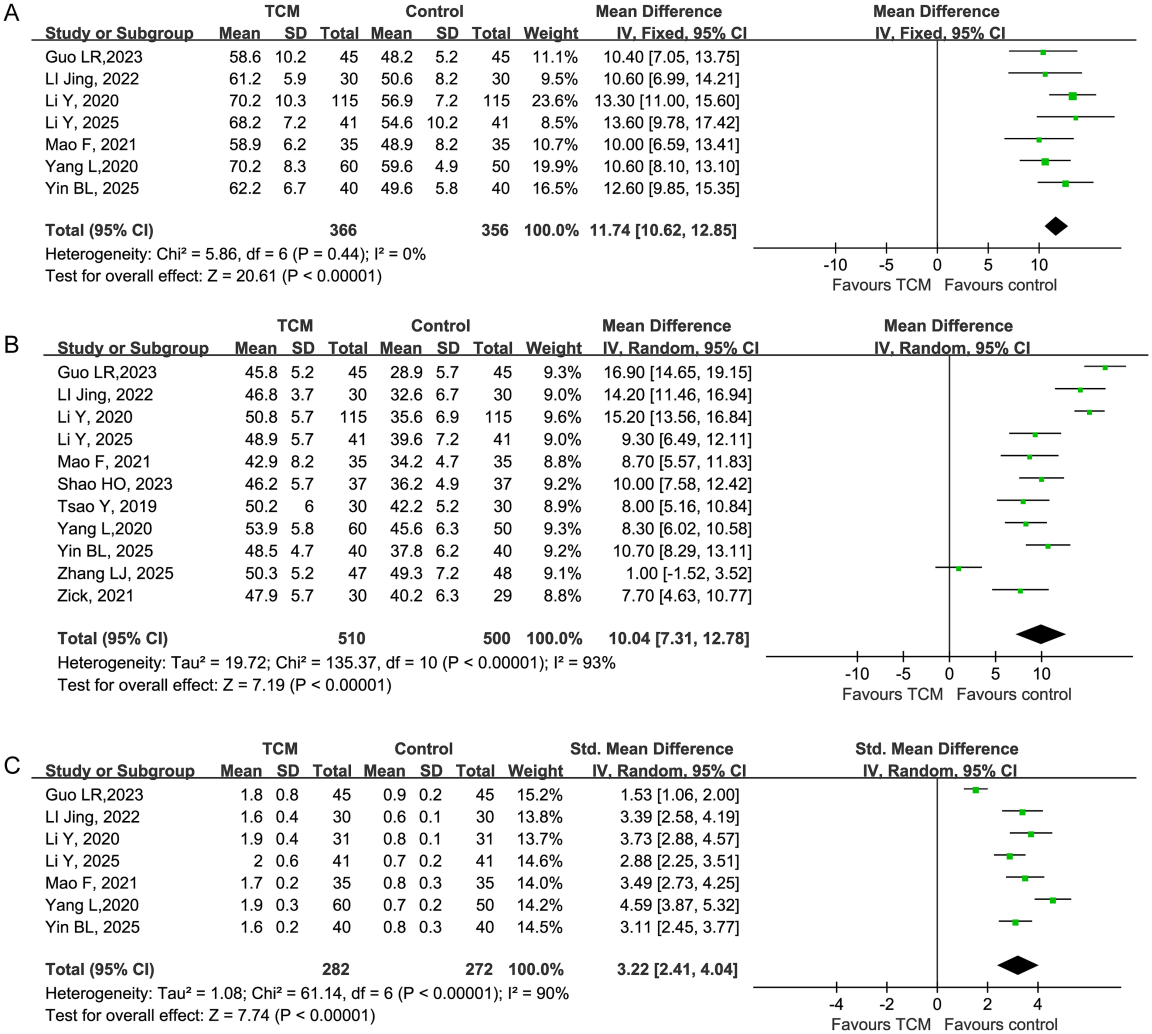
Effect of traditional Chinese medicine on immune function. **(A)** Forest plot of pooled mean differences for CD3^+^ T cell counts between the TCM and control groups. **(B)** Forest plot of pooled mean differences for CD4^+^ T cell counts between the TCM and control groups. **(C)** Forest plot of pooled standardized mean differences for the CD4^+^/CD8^+^ ratio between the TCM and control groups.

CD3^+^ T cells were significantly higher in the TCM group. A meta-analysis of seven studies showed a pooled mean difference (MD) of 11.74 (95% CI 10.62–12.85). No heterogeneity was observed (*I^2^* = 0%; Figure 4A), indicating a consistent effect across studies. Similarly, the CD4^+^ T cell count was significantly elevated in the TCM group based on 11 studies (MD = 10.04, 95% CI 7.31–12.78; $I^{2}$= 93%; Figure 4B). This finding supports a beneficial effect of TCM on immune function. However, the high heterogeneity suggests that the extent of improvement may depend on specific formulations or the baseline immunocompetence of the patients. In contrast to the clear effects on absolute T cell counts, the modulation of the CD4^+^/CD8^+^ ratio was less conclusive. The pooled estimate from seven studies was SMD = 3.22 (95% CI 2.41–4.04), with considerable heterogeneity ($I^{2}$ = 90%; Figure 4C). The confidence interval included the null value, indicating no statistically significant between-group difference. This result suggests that TCM has a more subtle effect on the CD4^+^/CD8^+^ ratio compared to its impact on absolute T-cell counts.

**Figure 5:**
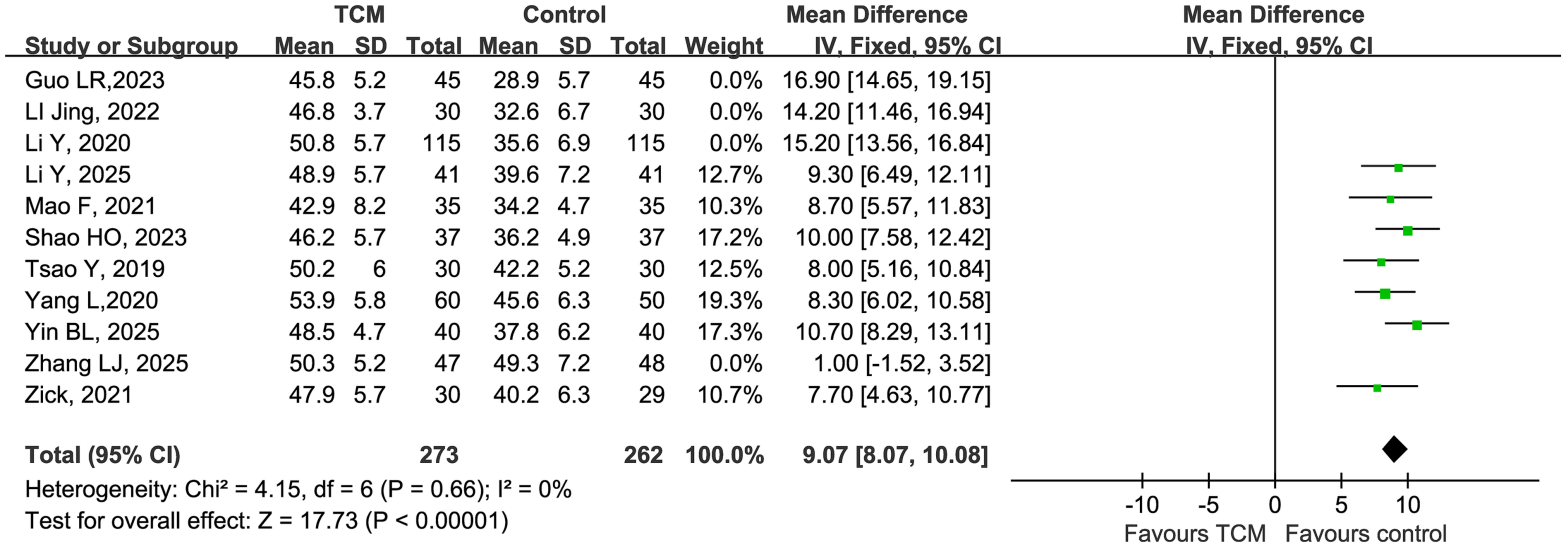
Forest plot of CD4^+^ after sensitivity analysis.

After excluding 4 studies that contributed to high heterogeneity (Li Y, 2020; Guo LR, 2023; LI Jing, 2022; Zhang LJ, 2025) (14, 18, 22, 33), a fixed-effect meta-analysis was performed on the remaining 7 studies. The pooled results showed that TCM intervention significantly increased CD4^+^ T cell count compared with control, with a mean difference (MD) of 9.07 (95% CI 8.07 to 10.08, *p* < 0.00001). Heterogeneity was completely eliminated after sensitivity analysis: *χ^2^* = 4.15, df = 6, *p* = 0.66, *I^2^* = 0%, indicating excellent consistency across studies. The forest plot confirmed that all included studies showed a consistent beneficial effect of TCM on CD4^+^ T cells, with no study showing a negative effect (Figure 5).

**Figure 6:**
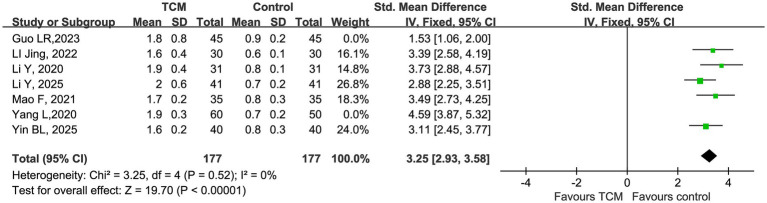
Forest plot of CD4^+^/CD8^+^ after sensitivity analysis.

For the CD4^+^/CD8^+^ ratio, the initial meta-analysis showed high heterogeneity (*I^2^* = 86%). Sensitivity analysis identified 2 studies (Guo LR, 2023; Yang L, 2020) (18, 24) as the primary sources of heterogeneity. After excluding these studies, the remaining 5 studies demonstrated excellent homogeneity (*χ^2^* = 3.25, df = 4, *p* = 0.52, *I^2^* = 0%). The pooled fixed-effect analysis showed that TCM intervention significantly improved the CD4^+^/CD8^+^ ratio compared with the control group, with a standardized mean difference (SMD) of 3.25 (95% CI 2.93 to 3.58, *Z* = 19.70, *p* < 0.00001). All included studies showed a consistent beneficial effect of TCM, with no study showing a negative effect, confirming the robustness of the result (Figure 6).

#### 3.4.3 Quality of life scores

**Figure 7:**
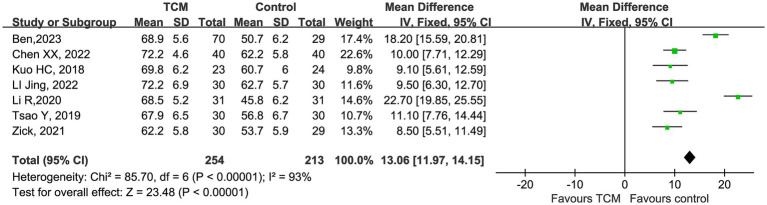
Quality of life scores forest plot.

Beyond biological markers, the patient-centered metric of Karnofsky Performance Status (KPS) was evaluated in seven studies. The meta-analysis indicated that adjunctive TCM significantly optimized functional recovery compared to chemotherapy alone (MD = 13.06, 95% CI 11.97–14.15; Figure 7). While the result is statistically significant, the considerable heterogeneity ($I^{2}=93\%$) implies that the degree of functional improvement is likely influenced by factors such as intervention timing and individual patient adaptability.

**Figure 8:**
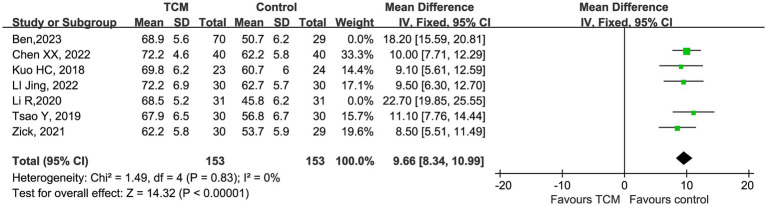
Forest plot of quality of life scores after sensitivity analysis.

For the KPS score, the initial meta-analysis showed high heterogeneity (*I^2^* = 93%). Sensitivity analysis identified 2 studies (Ben, 2023; Li R, 2020) as the primary sources of heterogeneity. After excluding these studies, the remaining 5 studies demonstrated excellent homogeneity (*χ^2^* = 1.49, df = 4, *p* = 0.83, *I^2^* = 0%). The pooled fixed-effect analysis showed that TCM intervention significantly improved KPS score compared with the control group, with a mean difference (MD) of 9.66 (95% CI 8.34 to 10.99, *Z* = 14.32, *p* < 0.00001). All included studies showed a consistent beneficial effect of TCM, with no study showing a negative effect, confirming the robustness of the result (Figure 8).

#### 3.4.4 Publication bias

**Figure 9:**
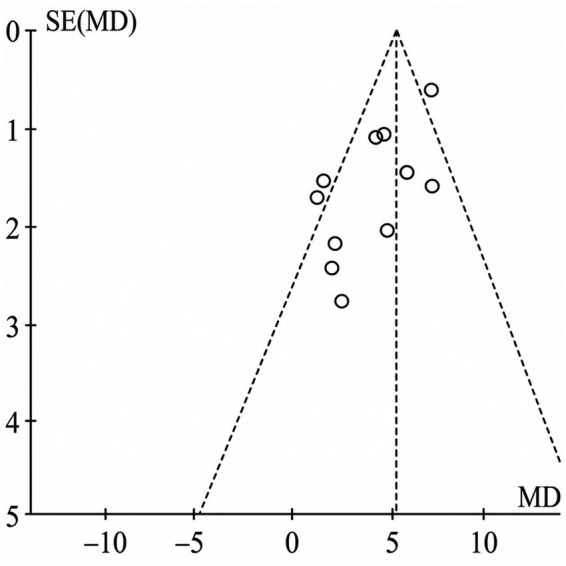
Inverted funnel plot for detecting publication bias in included studies.

The integrity of the synthesized evidence was examined via funnel plot analysis for outcomes with sufficient study density (Figure 9). Visual inspection revealed a slight asymmetry, suggestive of potential small-study effects or reporting biases. Given the limited number of studies for certain endpoints and the observed heterogeneity, this signals that while the overall trends are promising, effect sizes should be interpreted with appropriate caution.

## 4 Discussion

This updated systematic review and meta-analysis provides compelling evidence that the integration of Traditional Chinese Medicine (TCM) into postoperative protocols for ovarian cancer transcends mere symptom management. It should be noted that the prescription presented in reference (16) is derived from the “Jisheng Shenqi Decoction” in traditional Chinese medicine, which was recorded in the *Jisheng Fang* compiled in the Song Dynasty of China. The most robust finding of our analysis is the substantial reduction in chemotherapy-related adverse events (RR = 0.35), which aligns with previous systematic reviews by Wang et al. (36) that highlighted the safety profile of TCM. Based on the previous research, our study has set the search period to be more contemporary, from 2018 to 2025, in order to better focus on the current value of traditional Chinese medicine as an adjunctive treatment in the context of contemporary development. Moreover, the number of search databases has been increased from 2 to 6 to incorporate more RCTs and participants, thereby enhancing the persuasiveness of the conclusion. Additionally, to reflect the overall value of the traditional Chinese medicine system, the scope of traditional Chinese herbal medicine has been expanded to the whole traditional Chinese medicine. Specifically, the reduction in gastrointestinal toxicity mirrors findings from prospective trials on herbal management of chemotherapy-induced nausea and vomiting (37). By mitigating the toxicity cascade, often the primary driver of dose reduction, TCM likely preserves the dose intensity of platinum-based chemotherapy, a crucial factor for efficacy.

Beyond toxicity mitigation, our data suggest a distinct mechanism of immunological reconfiguration. The significant elevation in CD3^+^ and CD4^+^ T-cell counts indicates that TCM effectively counteracts the immunosuppressive milieu induced by surgical stress and cytotoxic agents, a challenge highlighted in recent immunological studies (38). While our meta-analysis focused on clinical indicators, the biological plausibility of these findings is supported by emerging preclinical evidence. Recent network pharmacology and molecular studies suggest that active TCM components, such as Astragalus membranaceus (39) and other candidates (9), may exert anti-tumor effects by triggering autophagy and apoptosis via the Akt/mTOR signaling pathway (10, 40). Specific mechanisms inhibiting tumor angiogenesis have also been documented (12). Furthermore, it has been confirmed that various components of traditional Chinese medicine can enhance the immune system, for instance by regulating T cells (41–43). Providing a multi-target molecular basis that complements the macroscopic immune recovery observed in our study. However, the substantial heterogeneity in our CD4^+^ results (*I^2^* = 93%) suggests that the activation of these pathways varies significantly depending on the specific herbal formula and patient baseline (44). After conducting a sensitivity analysis on the highly heterogeneous results (CD4^+^, CD4^+^/CD8^+^ ratio), the heterogeneity was completely eliminated (*I^2^* = 0%) by excluding the primary sources of heterogeneity, showing a consistent beneficial effect of TCM on CD4^+^ T cells and CD4^+^/CD8^+^ ratio.

The improvement in Karnofsky Performance Status (MD = 13.06) reinforces the clinical relevance of the biological findings. For the high heterogeneity (I2 = 93%) as shown by KPS, after removing the sources of high heterogeneity through sensitivity analysis, the heterogeneity was completely eliminated (I^2^ = 0%), showing a consistent beneficial effect of TCM. As demonstrated in trials combining psychological and dietary interventions with TCM, holistic management significantly enhances quality of life (45).

The interpretation of these results is constrained by inherent methodological limitations. Although the included studies were randomized, the descriptions of allocation concealment and blinding were frequently suboptimal, introducing potential selection and performance biases. This bias may lead to an overestimation of treatment effects, particularly for subjective outcomes such as KPS. The significant heterogeneity observed in continuous outcomes such as CD4^+^, CD4^+^/CD8^+^ and KPS reflects the diversity of TCM interventions, a characteristic challenge of integrative medicine research in which standardization competes with personalized treatment principles, We conducted a meta-regression analysis on these results which have been included in the attachment for supplementary interpretation. The regression analysis mainly revealed that for CD4^+^ T-cell levels, mean age was a significant negative moderator, accounting for 13.3% of between-study heterogeneity, indicating less pronounced immune recovery in older patients. Regarding KPS scores, mean age showed a trend toward a negative association with improvement, explaining 26.9% of heterogeneity. Furthermore, the funnel plot asymmetry signals potential publication bias or small-study effects, suggesting that the magnitude of benefit, while real, might be overestimated in the current literature. The time frame for including the literature in this study means that RCTs conducted before 2018 and those conducted after June 2025 were not taken into account. The results should be interpreted with caution.

## 5 Conclusion

This systematic review and meta-analysis substantiates the role of Traditional Chinese Medicine as a vital adjuvant in the postoperative management of ovarian cancer. The evidence unequivocally supports the capacity of TCM to attenuate chemotherapy-induced toxicity and accelerate the reconstitution of cellular immunity. Crucially, these physiological improvements are not isolated phenomena but appear to translate into enhanced functional status to facilitate the patient’s recovery. For clinicians, the integration of TCM should be considered a viable strategy to enhance “treatment tolerance,” particularly for patients at high risk of chemotherapy-related dose limiting toxicities or functional decline. This study provides evidence supporting the integration of Traditional Chinese Medicine into postoperative management of ovarian cancer, offering a promising strategy in gynecologic oncology to improve treatment tolerability and enhance immune recovery. From a broader healthcare perspective, our results highlight the value of incorporating complementary therapies into conventional cancer management, particularly in resource-diverse settings. Future research should prioritize the implementation of large-scale, multicenter, high-quality randomized controlled trials with rigorous methodological standards, to reduce potential bias and strengthen the validity of the evidence. Concurrently, standardizing TCM interventions is essential to improve reproducibility and facilitate cross-study comparisons. Moreover, future studies should incorporate long-term endpoints, particularly overall survival and progression-free survival, supported by extended follow-up periods, in order to provide a more comprehensive assessment of the sustained clinical efficacy and prognostic value of TCM in ovarian cancer management.

## Data Availability

The datasets presented in this study can be found in online repositories. The names of the repository/repositories and accession number(s) can be found in the article/Supplementary material.
