# Supplementary material for: Toxicity reduction and immune reconstitution with adjuvant traditional Chinese medicine for postoperative ovarian cancer: a systematic review and meta-analysis
Source: Front Med (Lausanne). 2026 Apr 30;13:1809880. doi: 10.3389/fmed.2026.1809880 (PMC13171591; doi:10.3389/fmed.2026.1809880)
Supplement: Supplementary file 1 [file Table_1.docx]

***Supplementary Material***

**electronic search strings**

**1. PubMed**

"Ovarian Neoplasms"[MeSH Terms] OR "ovarian cancer"[Title/Abstract] OR "ovarian tumor"[Title/Abstract] OR "ovarian carcinoma"[Title/Abstract] OR "ovary cancer"[Title/Abstract]

"Medicine, Chinese Traditional"[MeSH Terms] OR "Drugs, Chinese Herbal"[MeSH Terms] OR "traditional Chinese medicine"[Title/Abstract] OR "Chinese herbal medicine"[Title/Abstract] OR "TCM"[Title/Abstract] OR "integrative medicine"[Title/Abstract]

"Chemotherapy, Adjuvant"[MeSH Terms] OR "Antineoplastic Agents"[MeSH Terms] OR "Platinum Compounds"[MeSH Terms] OR "chemotherapy"[Title/Abstract] OR "platinum-based"[Title/Abstract]

"Randomized Controlled Trial"[Publication Type] OR "randomized controlled trial"[Title/Abstract] OR "randomized"[Title/Abstract] OR "clinical trial"[Title/Abstract]

#1 AND #2 AND #3 AND #4

**2. Cochrane Library**

[MeSH descriptor: [Ovarian Neoplasms]] OR "ovarian cancer":ti,ab,kw OR "ovarian tumor":ti,ab,kw OR "ovarian carcinoma":ti,ab,kw

[MeSH descriptor: [Medicine, Chinese Traditional]] OR [MeSH descriptor: [Drugs, Chinese Herbal]] OR "traditional Chinese medicine":ti,ab,kw OR "Chinese herbal medicine":ti,ab,kw OR "TCM":ti,ab,kw

[MeSH descriptor: [Chemotherapy, Adjuvant]] OR [MeSH descriptor: [Antineoplastic Agents]] OR "chemotherapy":ti,ab,kw OR "platinum-based":ti,ab,kw

[MeSH descriptor: [Randomized Controlled Trial]] OR "randomized controlled trial":ti,ab,kw OR "randomized":ti,ab,kw

#1 AND #2 AND #3 AND #4

**3. CNKI**

主题=('卵巢癌'+'卵巢肿瘤'+'卵巢恶性肿瘤')*('中医药'+'中药'+'中西医结合'+'参芪扶正'+'艾迪'+'康莱特')*('化疗'+'铂类'+'术后')*('随机对照试验'+'随机分组'+'对照研究')

**4. VIP**

标题:(卵巢癌 OR 卵巢肿瘤 OR 卵巢恶性肿瘤) AND 关键词:(中医药 OR 中药 OR 中西医结合 OR 参芪扶正 OR 艾迪 OR 康莱特) AND 关键词:(化疗 OR 铂类 OR 术后化疗) AND (随机对照试验 OR 随机分组 OR 对照研究)

**5. SinoMed**

(卵巢癌[主题词] OR 卵巢肿瘤[主题词] OR 卵巢恶性肿瘤[自由词]) AND (中医药疗法[主题词] OR 中药疗法[主题词] OR 中西医结合疗法[主题词] OR 参芪扶正注射液[自由词] OR 艾迪注射液[自由词] OR 康莱特注射液[自由词]) AND (化学疗法[主题词] OR 铂类化合物[主题词] OR 术后化疗[自由词]) AND (随机对照试验[主题词] OR 临床试验[主题词] OR 随机分组[自由词])

**6. Wanfang**

主题:(卵巢癌 OR 卵巢肿瘤 OR 卵巢恶性肿瘤) AND 主题:(中医药 OR 中药 OR 中西医结合 OR 参芪扶正 OR 艾迪 OR 康莱特) AND 主题:(化疗 OR 铂类 OR 术后化疗) AND 主题:(随机对照试验 OR 随机分组 OR 对照研究)
