# Supplementary material for: Toxicity reduction and immune reconstitution with adjuvant traditional Chinese medicine for postoperative ovarian cancer: a systematic review and meta-analysis
Source: Front Med (Lausanne). 2026 Apr 30;13:1809880. doi: 10.3389/fmed.2026.1809880 (PMC13171591; doi:10.3389/fmed.2026.1809880)
Supplement: Supplementary file 2 [file Table_2.DOCX]

***Supplementary Material***

Meta-regression Analysis

Given the substantial heterogeneity observed in CD4⁺ (*I²* = 93%), CD4⁺/CD8⁺ ratio (*I²* = 86%), and KPS scores (*I²* = 93%), meta-regression analyses were conducted to explore potential sources of between-study variance (Table S1).

For CD4⁺ levels, the type of TCM intervention explained 21.5% of the heterogeneity (overall *P* = 0.038). Specifically, compared to TCM decoctions (reference group), compound preparations showed a significantly greater effect on increasing CD4⁺ levels (coefficient = 0.74, 95% CI 0.24−1.46; *P* = 0.026), whereas external therapies demonstrated a significantly smaller effect (coefficient = −1.25, 95% CI −2.82 to −0.71; *P* = 0.015). In contrast, intervention type was not a significant moderator for the CD4⁺/CD8⁺ ratio (overall *P* = 0.428, R² = 14.4%) or KPS scores (overall *P* = 0.582, R² = 5.6%), suggesting that the heterogeneity in these outcomes is likely driven by other clinical or methodological factors not captured by intervention category alone

Meta-regression analyses for the continuous covariates (mean age and treatment duration) yielded the following results: For CD4⁺ T-cell levels, mean age was a significant negative moderator (Coeff. = -0.32, 95% CI -0.58 to -0.06; *P* = 0.018), accounting for 13.3% of between-study heterogeneity, indicating less pronounced immune recovery in older patients. Treatment duration was not a significant predictor (P = 0.352), explaining only 8.7% of the variance. For the CD4⁺/CD8⁺ ratio, neither mean age (Coeff. = 0.003, 95% CI -0.01 to 0.02; P = 0.582, R² = 6.4%) nor treatment duration (Coeff. = -0.03, 95% CI -0.08 to 0.02; P = 0.218, R² = 17.8%) showed a significant association, suggesting variability arises from other factors. Regarding KPS scores, mean age showed a trend toward a negative association with improvement (Coeff. = -0.08, 95% CI -1.12 to 0.96), explaining 26.9% of heterogeneity (Figure S1).

Table S1. Meta-regression Analysis of TCM Intervention Types on High-Heterogeneity Outcomes

| Outcome | Comparison | Studies（n） | Coefficient | 95% CI | P-value | R² |
| --- | --- | --- | --- | --- | --- | --- |
| CD4⁺ | Overall |  | — | — | 0.038* | 21.5% |
|  | Decoction (Ref) | 6 | — | — | — |  |
|  | External Therapy | 2 | -1.25 | (−2.82, -0.71) | 0.015* |  |
|  | Compound | 2 | 0.74 | (0.24, 1.46) | 0.026* |  |
|  | Injection | 1 | 1.48 | (−0.52, 3.72) | 0.182 |  |
| CD4⁺/CD8⁺ | Overall |  | — | — | 0.428 | 14.4% |
|  | Decoction (Ref) | 4 | — | — | — |  |
|  | Compound | 2 | 1.08 | (−1.15, 2.31) | 0.482 |  |
|  | Injection | 1 | 1.32 | (−0.48, 3.24) | 0.106 |  |
|  | External Therapy | 0 | — | — | — |  |
| KPS | Overall |  | — | — | 0.582 | 5.6% |
|  | External Therapy (Ref) | 6 | — | — | — |  |
|  | Compound | 1 | 2.34 | (−1.12, 3.80) | 0.398 |  |

CI, confidence interval; KPS, Karnofsky Performance Status; R², proportion of between-study variance explained; TCM, traditional Chinese medicine; vs., versus. Ref, Reference group


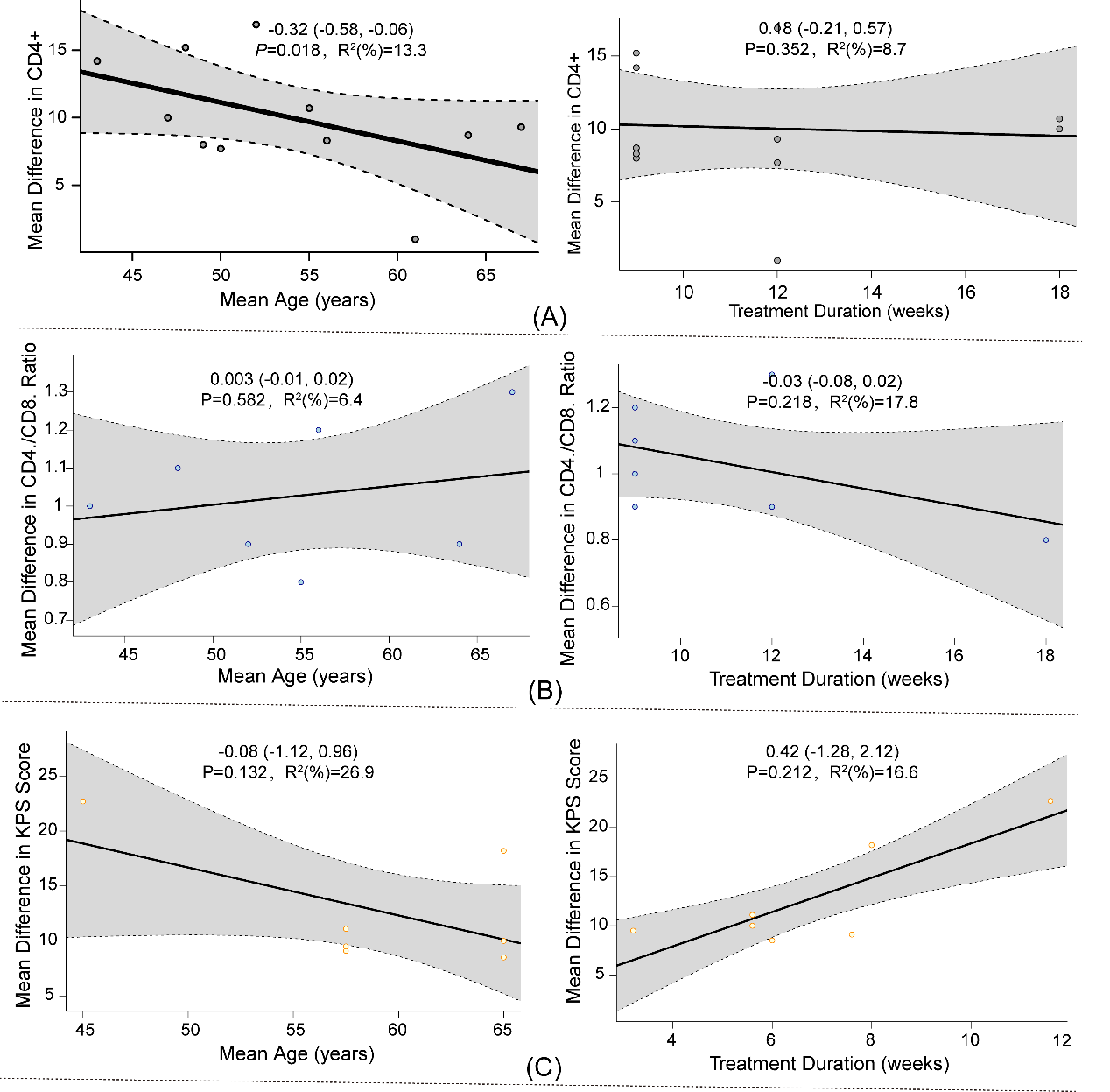


Figure S1. Meta-regression analysis of potential moderators (mean patient age and treatment duration) for between-study heterogeneity in immune function and Karnofsky Performance Status (KPS) improvements. Panels show the relationship of these moderators with changes in (A) CD4⁺ T cell levels, (B) CD4⁺/CD8⁺ ratio, and (C) KPS score. Solid black lines indicate fitted regression lines, with gray shading representing the 95% confidence interval.
